# Changes to bone mineral density, the trabecular bone score and hip structural analysis following parathyroidectomy: a case report

**DOI:** 10.1186/s12882-020-02168-y

**Published:** 2020-11-26

**Authors:** Raymond Lin, Mirna Vucak-Dzumhur, Grahame J. Elder

**Affiliations:** 1 Department of Renal Medicine, Westmead Hospital, Westmead, NSW 2145 Australia; 2 University of Notre Dame Medical School, Darlinghurst, NSW Australia; 3 Western Sydney University, Campbelltown Campus, Campbelltown, NSW Australia; 4 Garvan Institute of Medical Research, Osteoporosis and Bone Biology Division, Darlinghurst, NSW Australia

**Keywords:** Hyperparathyroidism, Parathyroidectomy, Bone mineral density, Hip structural analysis

## Abstract

**Background:**

Reduction in bone mineral density (BMD) measured by dual-energy X-ray absorptiometry (DXA) occurs in secondary hyperparathyroidism associated with chronic kidney disease. BMD generally increases following parathyroidectomy, however longitudinal changes to other DXA-derived parameters, the trabecular bone score (TBS) and hip structural analysis (HSA), have not been described. Postoperative calcium requirements and positive calcium balance raise concerns for an increased risk of vascular calcification. This case illustrates the dramatic increase in BMD that can follow parathyroidectomy in a patient on dialysis, and for the first time demonstrates improvements to HSA parameters and to the TBS.

**Case presentation:**

A 30-year old woman on haemodialysis underwent subtotal parathyroidectomy for secondary hyperparathyroidism. She developed a post-operative ‘hungry bone syndrome’ requiring substantial calcium and calcitriol supplementation. Six months post-parathyroidectomy, BMD increased by 42% at the lumbar spine, 30% at the femoral neck and 25% at the total proximal femur, with increases sustained over the following 18 months. The TBS increased by 8%. HSA showed a 63% increase in femoral neck cortical thickness and 38% reduction in the buckling ratio, consistent with increased femoral neck stability. The abdominal aortic vascular calcification score (0–24) increased from zero 8-years pre-parathyroidectomy to 2/24 at 18-months post-parathyroidectomy.

**Conclusion:**

BMD losses incurred by secondary hyperparathyroidism recover rapidly after parathyroidectomy, particularly at sites of trabecular bone. Bone architectural parameters, measured as the TBS and by HSA, also improve. Greater BMD gains may be associated with higher post-operative calcium requirements. While bone is the major reservoir for post-parathyroidectomy calcium supplementation, positive calcium balance may contribute to vascular calcification risk.

## Background

Many patients with end-stage kidney disease (ESKD) and secondary hyperparathyroidism have reduced bone mineral density (BMD), particularly in regions consisting primarily of cortical bone, and with relative sparing of trabecular bone. Following parathyroidectomy, BMD generally increases, but longitudinal changes to the trabecular bone score, and to parameters measured by hip structural analysis have not been described. In addition, post-operative positive calcium balance caused by requirements for massive supplementation with calcium and calcitriol or its analogues, raises concern for the risk of vascular calcification.

## Case presentation

A 30-year old woman underwent subtotal parathyroidectomy in September 2018, with removal of three parathyroid glands and auto-transplantation of half an excised gland to the sternocleidomastoid. Her background included ESKD secondary to atypical haemolytic uraemic syndrome, with haemodialysis from 2009 until a living-related kidney transplant in 2010 at the age of 22. However, disease recurrence resulted in graft failure, nephrectomy and recommencement of home haemodialysis in 2014.

**Fig. 1:**
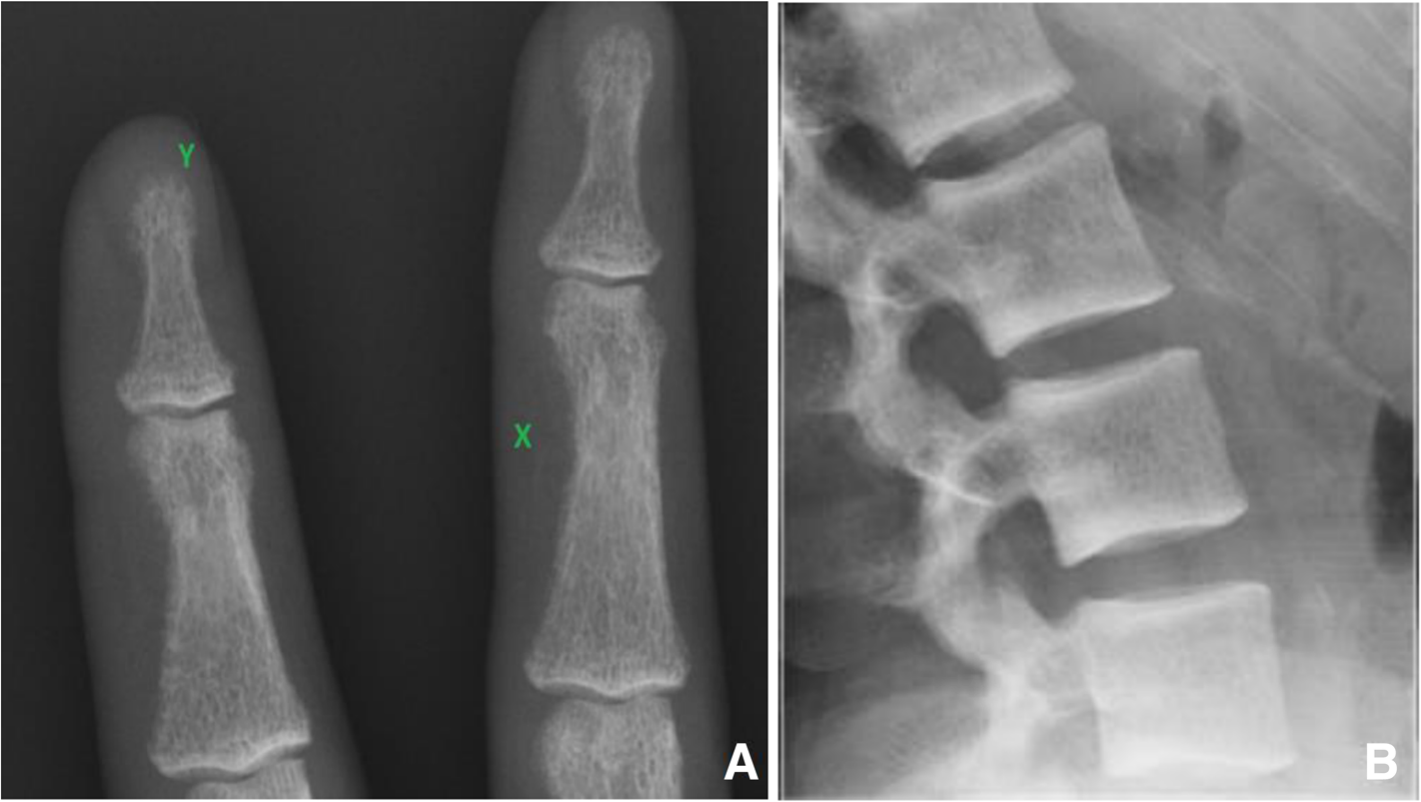
**a** Hand X-ray demonstrating cortical erosion of the radial aspect of the right middle phalanges (X) and loss of distal tuft of the right index finger (Y). **b** Rugger-Jersey spine with an alternating sclerotic-lucent appearance is characteristic of hyperparathyroidism. Sclerosis occurs towards the vertebral endplates, which are a bilayer of porous, fused trabecular bone, which allows for nutrient transport to the intervertebral disc, and cartilage with horizontally oriented collagen fibres. Linear calcification is visible in the abdominal aorta opposite the L4 vertebra, together with a fleck opposite L3

In 2015, 1 year after recommencing dialysis, her serum parathyroid hormone (PTH) was 111.3 pmol/L (normal range 1.6–7.5 pmol/L), rising to 273 pmol/L in 2017. Hand X-rays showed subperiosteal resorption consistent with hyperparathyroidism (Fig. 1a). In September 2018 her medications included calcitriol 0.25 μg alternating with 0.5 μg daily, sevelamer 2400 mg three-times daily and risedronate 35 mg weekly, which was discontinued 3 weeks prior to her parathyroidectomy in September 2018. Despite pre-operative loading with calcitriol 2.25 μg daily for 3 days, she developed a postoperative ‘hungry bone syndrome’ requiring intermittent intravenous calcium for up to 17 days, and daily doses of 8 μg calcitriol and 9600 mg calcium carbonate at discharge. She was also dialysed against an ionised calcium dialysate of 1.7 mmol/L for 6 months. Three months post-parathyroidectomy her daily dose of calcitriol had reduced to 1.5 μg and calcium carbonate 600 mg, which was maintained until a second kidney transplant in February 2020. Values of serum calcium, phosphate, ALP, PTH and bone turnover markers are shown in Table 1.

**Table 1:** Laboratory values before and after parathyroidectomy in September 2018, and kidney transplantation in February 2020. Patient was on haemodialysis from 2014 to February 2020

	Nov 2017	June 2018 3 months Pre-PTx	Dec. 2018 3 months Post-PTx	Feb. 2020 Pre-Tx	May 2020 3 months Post-Tx
Corrected Calcium (2.15–2.55 mmol/L)	2.23	2.29	2.37	2.47	2.32
Phosphate (0.75–1.50 mmol/L)	1.93	1.75	0.92	1.50	0.96
Parathyroid Hormone (1.6–7.5 pmol/L)	273.3	267.9	18.0	2.8	9.8
Alkaline Phosphatase (30–110 U/L)	353	503	41	43	38
CTX (100–700 ng/L)	–	–	–	879	472
P1NP (15–90 μg/L)	–	–	–	127	48

*PTx* Parathyroidectomy, *Tx* Kidney transplantation, *CTX* C-Terminal Collagen Type 1 Telopeptide Crosslinks, *P1NP* Procollagen Type 1 N-Terminal Propeptide

Her BMD was measured by dual-energy X-ray absorptiometry (DXA) at her first transplant in 2010, 8 months prior to parathyroidectomy in January 2018, 6 months after parathyroidectomy in March 2019 and at her 2nd transplant in March 2020 (Table 2). The trabecular bone score (TBS) and hip structural analysis (HSA) were also measured from DXA pre- and post-parathyroidectomy (Table 2). TBS is an indirect measure of cancellous bone microarchitectural integrity, and HSA provides information on cortical thickness at sites around the hip, and the buckling ratio (BR), calculated as the femoral neck radius divided by the femoral neck cortical thickness. Higher BR values are consistent with increased femoral neck instability.

**Table 2:** Bone mineral densities (BMD), trabecular bone score (TBS) and hip structural analysis parameters over time. Due to her age, T and Z-scores are identical

	August 2010 ^**a**^	January 2018 ^**b**^ 8 months Pre-PTx	March 2019 ^**b**^ 6 months Post-PTx	March 2020 ^**b**^ 18 months Post-PTx
**Bone Mineral Density**				
LS BMD (g/cm^2^)	1.06	1.02	1.44	1.38
LS T-score	−0.3	−1.4	+ 1.8	+ 1.3
% Change		Baseline	+ 42%	+ 38%
TPF BMD (g/cm^2^)	0.94	0.84	1.05	1.16
TPF T-score	−0.2	−1.6	0	+ 0.8
% Change		Baseline	+ 25%	+ 36%
FN BMD (g/cm^2^)	0.94	0.81	1.05	1.16
FN T-score	0.0	−1.6	+ 0.2	+ 1.1
%Change		Baseline	+ 30%	+ 43%
UD-R BMD (g/cm^2^)	0.28^c^	0.27	0.31	0.34
UD-R T-score	−0.9^c^	−4.3	−3.4	−2.8
% Change		Baseline	+ 13%	+ 23%
1/3-R BMD (g/cm^2^)	0.76^c^	0.62	0.59	0.63
1/3-R T-score		−2.9	−3.3	−2.9
% Change		Baseline	−4.8%	+ 1.6%
TBS		1.56	1.68	1.56
TBS T-score		+ 0.8	+ 2.0	+ 0.8
% Change		Baseline	+ 8%	0%
**Hip Geometry and Hip Structural Analysis**				
Femoral neck cortical width (mm) (% Change)		4.6	7.5 (+ 63%)	6.9 (+ 50%)
Femoral calcar width (mm) (% Change)		3.5	4.1 (+ 17%)	4.2 (+ 20%)
Femoral shaft width (mm) (% Change)		5.5	5.6 (+ 1.8%)	6.5(+ 18%)
Buckling Ratio (% Change)		3.4	2.1 (−38%)	2.2 (−35%)
Body Mass Index (kg/m^2^)		26.71	26.80	25.64

^a^Scan performed on Norland XR800

^b^Scan performed on Lunar iDXA

^c^Norland measures combined ultradistal radius and ulnar BMD. 1/3 radius T-score was unavailable

By 6 months post-parathyroidectomy, BMD at the lumbar spine increased by 42% and at the hip by 25–30%. The TBS T-score increased from + 0.8 to 2. Using HSA, the femoral neck cortical thickness increased by 63% and the buckling ratio fell by 38%. Her BMI remained stable throughout. The abdominal aorta calcification score (AACS) (0–24) was measured by the Kauppila semiquantitative scoring method from a lateral spine X-ray that included the abdominal aorta [1]. The score was zero in 2011 and 2/24 in March 2020 (Fig. 1b). Radiological changes of Rugger-Jersey spine were present in 2011 and were more prominent in 2020 (Fig. 1b).

## Discussion and conclusions

This patient’s BMD declined between 2010 and January 2018, with the likely nadir immediately pre-parathyroidectomy. Although moderately elevated circadian PTH values are anabolic to bone, persistently high values increase osteoblast expression of RANK ligand and reduce levels of osteoprotegerin, the decoy receptor for RANK ligand, which increases osteoclastogenesis and osteoclast activity. In addition, high PTH levels cause proliferation of pre-osteoblasts, which do not mature due to a lack of bone morphogenic protein 7, a growth factor primarily of renal origin [1]. The effects of hyperparathyroidism differ by site [2], with exclusively cortical sites such as the metacarpals and 1/3 distal radius often showing greatest BMD loss. However, using peripheral quantitative computed tomography, deterioration of trabecular bone has also been demonstrated in dialysis patients with hyperparathyroidism and patients with primary hyperparathyroidism [3, 4]. In this patient, predominantly cortical forearm sites were most severely affected prior to parathyroidectomy, with the 1/3 radius T-score − 2.9 and ultradistal radius T-score − 4.3, while the hip and vertebrae showed lesser BMD reductions.

Within 6 months of parathyroidectomy, BMD gains up to 42% occurred at the spine and hip, with a smaller but significant improvement at the ultradistal radius and no significant change at the 1/3 radius, a site of cortical bone. After parathyroidectomy, the high surface area of trabecular bone facilitates mineralisation of former resorption sites, and secondary mineralisation due to reduced bone remodelling, whereas in cortical bone, the reduced cortical thickness and increased porosity are less readily repaired. In the lumbar spine, the TBS was high normal (TBS T-score + 0.8) prior to parathyroidectomy, suggesting that the trabecular network had not been severely compromised in this patient. The TBS showed an increase following parathyroidectomy, which returned to the baseline value over the subsequent year on dialysis.

Patients with primary and secondary hyperparathyroidism are recognised to have gains in BMD of 10–15% following parathyroidectomy [5, 6], and patients on haemodialysis have recorded gains of 7–23% [7]. The ‘hungry bone syndrome’ has also been associated with impressive BMD gains. In a case series of Indian patients with primary hyperparathyroidism, 46 of 51 patients developed post-operative hypocalcaemia, with BMD increases at 12 months of 106% (IQR 67–178%) at the spine and 133% (IQR 54–176%) at the hip [8]. The current case demonstrates that large increases in BMD may also occur in a patient on dialysis with severe secondary hyperparathyroidism and the ‘hungry bone syndrome’.

HSA uses two-dimensional hip DXA images to estimate bone geometry and bone strength. HSA has been shown to enhance hip fracture risk stratification and is comparable to CT-based HSA measurements [9, 10]. A recent study showed that HSA parameters are markedly abnormal in patients on dialysis [11], but longitudinal changes to HSA parameters have not been reported. This patient showed marked improvements in all hip parameters including femoral neck, calcar and shaft width, and a reduction in the buckling ratio after parathyroidectomy, indicative of improved femoral neck stability and possibly lower fracture risk.

The necessity for high dose calcium and vitamin D following parathyroidectomy and treatment of the ‘hungry bone syndrome’ raises concern for the development of vascular calcification due to a positive calcium balance [12]. This patient required ongoing high dose oral calcium and calcitriol and was dialysed against a high dialysate calcium for 6 months from the time of parathyroidectomy. The 2017 Kidney Disease Improving Global Outcomes (KDIGO) guidelines for Mineral and Bone Disorders suggest that patients identified with vascular calcification on a lateral abdominal radiograph be considered at highest cardiovascular risk [13] and recent data indicates that the AACS (0–24) also predicts cardiovascular risk and mortality after transplantation [14]. Over the period from 2011 to 2020, this patient’s AACS (0–24) increased from zero to 2/24. This small, but possibly significant rise may reflect her longer time on dialysis, increasing age, or hyperparathyroidism and markedly elevated ALP, which hydrolyses pyrophosphate, an endogenous inhibitor of calcification in blood vessels and bone. However, we cannot exclude a contribution from the positive calcium balance that occurred during treatment of her post-parathyroidectomy hypocalcaemia. For a patient with hypocalcaemia, even relatively low (1.25 mmol/L) calcium dialysate may result in positive calcium mass transfer from dialysate to blood [13] .

This patient’s clinical progress demonstrates that BMD losses associated with secondary hyperparathyroidism recover after parathyroidectomy, particularly at sites of trabecular bone. There may also be improvement in bone architectural parameters, because TBS and HSA parameters improved. Longitudinal changes to these parameters have not been previously reported in patients on dialysis following parathyroidectomy. BMD gains may be greater in patients who develop a ‘hungry bone syndrome’. Although bone is the major reservoir for calcium following parathyroidectomy, we cannot exclude the possibility of positive calcium balance potentiating risks for progression of vascular calcification.

## Data Availability

Not applicable.

## Abbreviations

AACS (0–24): Abdominal Aorta Calcification Score (0–24)
ALP: Alkaline Phosphatase
BMD: Bone Mineral Density
BR: Buckling Ratio
DXA: Dual-Energy X-ray Absorptiometry
ESKD: End-Stage Kidney Disease
HSA: Hip Structural Analysis
KDIGO: Kidney Disease Improving Global Outcomes
PTH: Parathyroid Hormone
TBS: Trabecular Bone Score
